# The relationship between psychological resilience and emotion regulation in Chinese adolescents: a psychological network analysis

**DOI:** 10.3389/fpsyg.2025.1552109

**Published:** 2025-11-19

**Authors:** Yingjun Xiang, Chengjun Lu, Shujuan Wei, Jiejing Hao, Xiaoya Sun, Ziyu Chen

**Affiliations:** 1 Shenzhen Futian Third People’s Hospital, Shenzhen Futian Center for Chronic Disease Control, Shenzhen, China; 2 Department of Public Health and Preventive Medicine, School of Medicine, Jinan University, Guangzhou, China; 3 Department of Medical Statistics and Epidemiology, School of Public Health, Sun Yat-sen University, Guangzhou, China; 4 Department of Psychiatry, Shenzhen Nanshan Center for Chronic Disease Control, Shenzhen, China

**Keywords:** psychological resilience, emotion regulation, China, adolescents, network analysis

## Abstract

**Background:**

Although psychological resilience and emotion regulation are crucial factors influencing mental health, network analysis studies examining their interrelationships in adolescents are scarce.

**Methods:**

A cross-sectional study was conducted in Shenzhen, China (October–November 2024). A total of 2,119 seventh-grade adolescents (Mean age = 12.25 ± 0.45 years; 1,101 males) were included. Network analysis was employed to model interactions among items measuring psychological resilience and emotion regulation. The network structure and gender differences were examined.

**Results:**

Females scored lower on psychological resilience dimensions and cognitive reappraisal, but higher on expressive suppression. The items “Setting goals to propel myself forward” (from resilience) and “Thinking positively when facing something unpleasant” (from emotion regulation) exhibited the highest node expected influence within their respective clusters. Bridge centrality analysis, supplemented by bootstrapped difference tests, identified the positive cognition item “Believing that everything has a positive side” as possessing statistically superior bridge strength, indicating it as the most robust connector between the two constructs. The network structure for females showed greater connectivity.

**Conclusion:**

This study provides a visualization of the complex network between psychological resilience and emotion regulation. The findings highlight the centrality of “positive cognition” as a critical and reliable intervention target, suggesting that fostering the ability to find positive aspects in adversity may most effectively enhance the entire resilience-emotion regulation system in adolescents.

## 1 Introduction

Adolescence, defined by the World Health Organization (WHO) as the period from ages 10 to 19, represents a critical developmental phase (WHO, 2024a). This study focuses on early adolescence (ages 12–14), which corresponds to the seventh grade in China—a stage characterized by substantial physical, cognitive, and social changes. Globally, mental health disorders affect approximately 14% of adolescents and contribute significantly to the global disease burden (WHO, 2024b). In China, the prevalence of high psychological distress among children and adolescents aged 9–18 is 31.6%, with this rate increasing with age (Chen et al., 2023). Understanding the factors that promote mental well-being during this vulnerable period is therefore essential. Psychological resilience and emotion regulation represent two critical, interrelated capacities that support positive adaptation.

### 1.1 Psychological resilience in adolescents

Psychological resilience is broadly conceptualized as the dynamic process of positively adapting to adversity (American, Psychological, and Association, 2017). It serves as a fundamental protective factor for mental health and is robustly associated with a reduced incidence of depressive and anxiety symptoms by mitigating the effects of stress. A large cross-sectional study of 6,019 high school students in China (Gong et al., 2020) found that psychological resilience significantly mediated the relationship between personality traits and depressive symptoms. Specifically, higher resilience reduced the risk associated with neuroticism by approximately 15%. Similarly, research involving 450 Taiwanese adolescents (Lee et al., 2021) revealed that resilience partially mediated the association between interpersonal relationships and depressive symptoms. This mediation effect was statistically significant, accounting for over 30% of the total effect.

### 1.2 Emotional regulation in adolescents

Emotion regulation refers to the process through which individuals modulate their emotional experiences and expressions (Gross and Thompson, 2007). It is commonly assessed using two core strategies: cognitive reappraisal and expressive suppression (Gross and John, 2003). Empirical studies have shown that emotion regulation plays an instrumental role in modulating depressive and anxiety symptoms among adolescents. A large longitudinal study by Gonçalves et al. (2019) demonstrated that difficulties in emotion regulation significantly predicted increases in depressive symptoms over time. Each standard deviation increase in baseline emotion regulation difficulties was associated with a 0.10 standard deviation increase in subsequent depressive symptoms. Additionally, a cross-sectional study of 430 Chinese high school students by Yu et al. (2022) found a negative correlation between cognitive reappraisal and anxiety symptoms.

### 1.3 The relationship between psychological resilience and emotion regulation among adolescents

Psychological resilience and emotion regulation are fundamentally interconnected constructs that exhibit a dynamic and reciprocal relationship, wherein each capacity enhances and facilitates the other (Troy and Mauss, 2011). This mutual reinforcement is particularly critical during adolescence—a period characterized by significant emotional and social development. Effective emotion regulation constitutes a core component of psychological resilience. Specifically, individuals with higher resilience demonstrate a greater capacity to modulate emotional responses when confronting challenges, thereby promoting faster recovery from negative emotional states (Gross and John, 2003). Conversely, the use of adaptive emotion regulation strategies, such as cognitive reappraisal, strongly predicts resilience. Adolescence represents a critical developmental window marked by substantial emotional, cognitive, and social changes, rendering this interplay especially relevant. For example, studies among Spanish adolescents have shown that cognitive regulation strategies, including positive reappraisal, predict resilience (Mestre et al., 2017). Neurobiological evidence further supports this, indicating that cognitive reappraisal facilitates effective emotion regulation through prefrontal modulation of amygdala reactivity—a key mechanism underlying resilient functioning (Goldin et al., 2008). Moreover, Kuhlman et al. (2021) assessed psychological well-being in high school students during the COVID-19 pandemic and found that adolescents with higher cognitive reappraisal capacity exhibited fewer psychiatric symptoms and maintained greater psychological resilience. Thus, the relationship between psychological resilience and emotion regulation is bidirectional and synergistic, with each capacity reinforcing the other.

### 1.4 Psychological network analysis

Traditional statistical methods in psychology (e.g., regression) often assume linear relationships and focus on net effects, potentially obscuring the complex interactions inherent in constructs such as psychological resilience and emotion regulation (Borsboom, 2017). Such dynamics likely involve feedback loops and nonlinear pathways that are not captured by conventional approaches. Psychological network analysis addresses these limitations by conceptualizing psychological phenomena as complex systems (van Borkulo et al., 2023). Within this framework, resilience and emotion regulation are viewed not as latent factors but as emergent properties arising from interactions among specific measurable components (nodes) (Epskamp et al., 2018). This methodology maps the entire web of pairwise interactions without pre-specifying independent or dependent variables, thereby allowing the identification of central features and bridge elements between constructs. As a result, it offers precise targets for intervention (Jones et al., 2021). Applying this approach to adolescents provides a novel means of visualizing interconnected psychological mechanisms and identifying leverage points for mental health promotion.

### 1.5 Gender differences in psychological resilience and emotion regulation among adolescents

Research reveals nuanced gender differences in psychological resilience and emotion regulation during adolescence. One study of Chinese university students indicated that males scored 12.3% higher than females in overall resilience, a difference linked to females’ greater susceptibility to stress related to the COVID-19 pandemic (Pan et al., 2024). In contrast, research conducted in Austria found that females scored 8–10% higher on certain resilience subscales, including personal competence and emotional control (Sojer et al., 2024). Regarding emotion regulation, females tend to use adaptive strategies such as cognitive reappraisal approximately 18% more frequently than males (Susan Nolen-Hoeksema et al., 2011). Neuroimaging studies further reveal differentiated neural correlates: males exhibit roughly 25% greater amygdala suppression during emotion regulation, suggesting more automated control, whereas females show about 15% greater activation in the ventral striatum, indicating enhanced reward-based processing (McRae et al., 2008). Cross-cultural research suggests that in collectivistic cultures (e.g., East Asian societies), gender-role socialization may amplify these disparities, as females are often encouraged to use expressive suppression more frequently (Matsumoto et al., 2008). In individualistic cultures, however, gender differences in the use of cognitive reappraisal strategies may be more pronounced (Chentsova-Dutton and Tsai, 2010). Future research should further examine the moderating role of cultural context in shaping gender differences in emotion regulation and psychological resilience.

### 1.6 The current study

There is a dearth of research using network analysis to examine the relationship between psychological resilience and emotion regulation in Chinese early adolescents. To address this gap, this study employed a network approach to delineate the interconnections between these constructs and identify pivotal bridge nodes. Based on existing literature, the following hypotheses were proposed:

*H1*: The items measuring psychological resilience and emotion regulation will form a cohesive and interconnected network structure. This is supported by evidence of the dynamic, mutually reinforcing relationship between resilience and emotion regulation (Troy and Mauss, 2011). Within this structure, we will exploratorily identify the most central nodes within each construct’s cluster.

*H2*: Nodes representative of positive cognition and cognitive reappraisal will serve as key bridges connecting the psychological resilience and emotion regulation networks. This prediction is supported by empirical evidence demonstrating that cognitive reappraisal significantly predicts psychological resilience in adolescent populations (Mestre et al., 2017), suggesting that the ability to find positive meaning and the strategy to positively reinterpret situations form a core pathway between these constructs.

*H3*: We will also exploratorily examine differences in network structure between male and female adolescents. Given the limited and inconsistent existing literature on gender differences in the network architecture of these constructs, we do not posit a specific *a priori* hypothesis regarding the nature of these differences.

## 2 Methods

### 2.1 Study design and participants

A cross-sectional study was conducted in Shenzhen, China, from October to November 2024. Participants were recruited from four junior high schools using a convenience sampling method. A total of 2,214 seventh-grade students were initially invited to participate.

#### 2.1.1 Inclusion and exclusion criteria

Inclusion criteria were: (1) current enrollment in one of the participating schools; (2) no self-reported history of neurological or psychiatric disorders; and (3) provision of voluntary informed consent. Exclusion was based on completion of the online questionnaire in less than 60 s, which was considered indicative of careless or non-viable responding.

#### 2.1.2 Sample characteristics

No missing data occurred in the online survey since all items were mandatory. However, 95 questionnaires were excluded based on the pre-defined exclusion criterion. The final analytical sample consisted of 2,119 participants (1,101 males and 1,018 females). The mean age of participants was 12.25 ± 0.45 years, with an age range of 12 to 14 years. It is important to note that detailed sociodemographic data (e.g., socioeconomic status, parental education) were not collected in the current study, which is acknowledged as a limitation in the Discussion section.

### 2.2 Procedure

All assessments were administered through the Shenzhen Futian District Mental Health Assessment System under the supervision of certified psychological educators. Participants were seated at individual desks to complete the questionnaires, minimizing peer interaction and ensuring confidentiality. Due to the need for participant identification and potential follow-up monitoring, the questionnaires were not anonymous. All students were explicitly informed of the principles of voluntary participation and response confidentiality. The study was conducted in accordance with the ethical principles of the Declaration of Helsinki (World Medical Association, 2025) and received approval from the Ethics Committee of the Shenzhen Futian Center for Chronic Disease Control (Approval no: 2024008). The study was conducted in accordance with the ethical principles of the Declaration of Helsinki (World Medical Association, 2025).

### 2.3 Measurements

#### 2.3.1 General demographic data

Sociodemographic information (age, sex) was collected using questionnaires designed by the research team.

#### 2.3.2 Resilience

The Resilience Scale for Chinese Adolescents (RSCA), as developed by Yue-Qin and Yi-Qun (2008), was utilized in this investigation. The scale consists of 27 items, categorized across five discrete dimensions: goal focus, emotion control, positive cognition, family support, and interpersonal assistance. The RSCA includes both positively and negatively worded items. Prior to analysis, all negatively worded items (e.g., RSCA26: ‘Unwilling to talk to others when feeling down’) were reverse-scored so that higher scores on all items indicate higher levels of psychological resilience. Participants were instructed to rate each item using a 5-point Likert scale, with higher composite scores reflecting greater resilience. The RSCA demonstrated a Cronbach’s alpha coefficient of *α* = 0.91 in this sample.

#### 2.3.3 Emotion regulation

The Emotional Regulation Questionnaire for Children and Adolescents (ERQ-CA), as devised by Gullone and Taffe (2012), is grounded in the emotional regulation process model (Gross, 1998). The ERQ-CA encompasses two prevalent emotion regulation strategies: cognitive reappraisal and expressive suppression. Cognitive reappraisal pertains to altering an individual’s emotional response through the reinterpretation of the emotional context, whereas expressive suppression pertains to the inhibition of the behavioral manifestation of an individual’s emotional response. The ERQ-CA is comprised of 10 items, each scored on a 5-point Likert scale, where 1 corresponds to “completely disagree” and 5 to “completely agree.” Scores on the cognitive reappraisal subscale range from 6 to 30, and on the expressive suppression subscale from 4 to 20, with higher scores reflecting greater employment of the respective strategies. The ERQ-CA exhibited a Cronbach’s alpha coefficient of *α* = 0.73 in this sample.

### 2.4 Statistical analysis

#### 2.4.1 Descriptive statistics

Descriptive statistics were computed using SPSS version 26.0. Sociodemographic characteristics were expressed as proportions, while dimensions of psychological resilience and emotion regulation were summarized using means and standard deviations. Relationships among all dimensions were assessed using Pearson correlation analysis.

#### 2.4.2 Network estimation

The network was estimated using a Graphical Gaussian Model (GGM) in the R environment. The GGM represents associations through partial correlation coefficients, which indicate the unique relationship between two nodes after controlling for all other variables. This approach is particularly suitable for identifying direct interactions between psychological constructs. To address high-dimensionality and obtain a sparse, interpretable network, we applied the graphical Least Absolute Shrinkage and Selection Operator (LASSO) regularization to the GGM. This method shrinks trivial edge weights to zero. The optimal level of sparsity was determined using the Extended Bayesian Information Criterion (EBIC), which balances model fit with complexity (Epskamp et al., 2012). This EBICglasso estimation was implemented via the qgraph package (version 1.6.9) (Epskamp et al., 2012), wherein edge thickness corresponds to the strength of the regularized partial correlation, with green and red edges denoting positive and negative associations, respectively.

#### 2.4.3 Network centrality

Centrality indices—including expected influence (EI) and strength—were computed to evaluate the relative importance of nodes within the network. These indices were calculated using the qgraph package (version 1.6.9) (Epskamp et al., 2012). Node strength is defined as the sum of the absolute values of all edge weights connected to a node. In contrast, expected influence represents the sum of edge weights without taking absolute values, reflecting the anticipated total impact of a node on the network (Epskamp et al., 2018). Due to the known instability of closeness and betweenness centrality in psychological networks, interpretation focused exclusively on EI and strength (Bringmann et al., 2019).

#### 2.4.4 Bridge centrality

Bridge centrality was determined using the R package networktools (version 1.2.3) (Jones, 2020). This package quantifies bridge strength and bridge expected influence (BEI), offering insights into how nodes mediate connections between distinct groups and their overall impact on the network (Jones et al., 2021). Unlike node centrality, which assesses the importance of nodes within a single group, bridge centrality specifically evaluates the role of nodes in connecting two separate groups, highlighting their critical function in linking disparate clusters. Following recommendations in the literature, only the top 20% of bridge nodes were retained for interpretation (Jones et al., 2021).

#### 2.4.5 Network accuracy and stability

The accuracy and stability of the estimated network were assessed using the bootnet package (Epskamp et al., 2018). We employed non-parametric bootstrapping (1,000 samples) to calculate 95% confidence intervals for edge weights, evaluating estimation precision. The stability of centrality indices (expected influence and strength) was examined using a case-dropping bootstrap approach, summarized by the correlation stability (CS) coefficient. A CS value greater than 0.50 is generally considered to indicate strong stability (Epskamp et al., 2018). Additionally, bootstrap difference tests were conducted to determine whether specific edges and centrality indices differed significantly from each other, ensuring the robustness of the network structure. Note that the interpretation of node and bridge centrality will primarily rely on the results of the bootstrapped difference tests, as these indicate the statistical robustness and significance of observed differences between nodes, whereas the raw centrality indices provide initial rankings within the sample.

#### 2.4.6 Network comparison

Given established gender differences in psychological resilience and emotion regulation, network characteristics were compared between male and female participants. Disparities in global and local connectivity were assessed using the Network Comparison Test (NCT), implemented via the R package NetworkComparisonTest (version 2.2.1) (van Borkulo et al., 2023). The analysis was performed with 1,000 permutations to evaluate the statistical significance of the observed differences.

## 3 Results

### 3.1 Descriptive statistics and correlation analysis

Descriptive statistics for each dimension of the study are presented in Table 1. With the exception of the family support, gender differences across all other dimensions were statistically significant. Male participants scored higher than females on the dimensions of goal focus, emotion control, positive cognition, interpersonal assistance, and cognitive reappraisal. Conversely, female participants exhibited higher scores on expressive suppression.

**Table 1:** Descriptive statistics of study dimensions (*N* = 2,119).

Dimension	Male	Female	*t*	*p*
	*M*(SD)	*M*(SD)		
Resilience				
Goal focus	3.45(0.89)	3.19(0.83)	6.92	<0.001
Emotional control	3.39(0.94)	2.98(0.93)	10.11	<0.001
Positive cognition	3.56(0.94)	3.29(0.82)	7.08	<0.001
Family support	3.46(0.88)	3.42(0.90)	1.04	0.30
Interpersonal assistance	3.36(0.99)	3.25(1.04)	2.45	0.01
Emotion regulation				
Cognitive reappraisal	3.13(0.91)	2.99(0.84)	3.50	<0.001
Expressive suppression	2.65(0.94)	2.75(0.88)	−2.368	0.02

As shown in Table 2, all seven dimensions demonstrated significant positive intercorrelations (*p* < 0.01).

**Table 2:** Correlations among all seven dimensions.

Dimension	1	2	3	4	5	6	7
GF	1						
EC	0.42**	1					
PC	0.64**	0.33**	1				
FS	0.39**	0.45**	0.30**	1			
IA	0.40**	0.58**	0.30**	0.48**	1		
CR	0.50**	0.33**	0.45**	0.27**	0.34**	1	
ES	−0.092**	−0.32**	−0.07**	−0.24**	−0.48**	0.12**	1

**p < 0.01, GF, goal focus; EC, emotional control; PC, positive cognition; FS, family support; IA, interpersonal assistance; CR, cognitive reappraisal; ES, expressive suppression.

### 3.2 Network estimation

**Figure 1:**
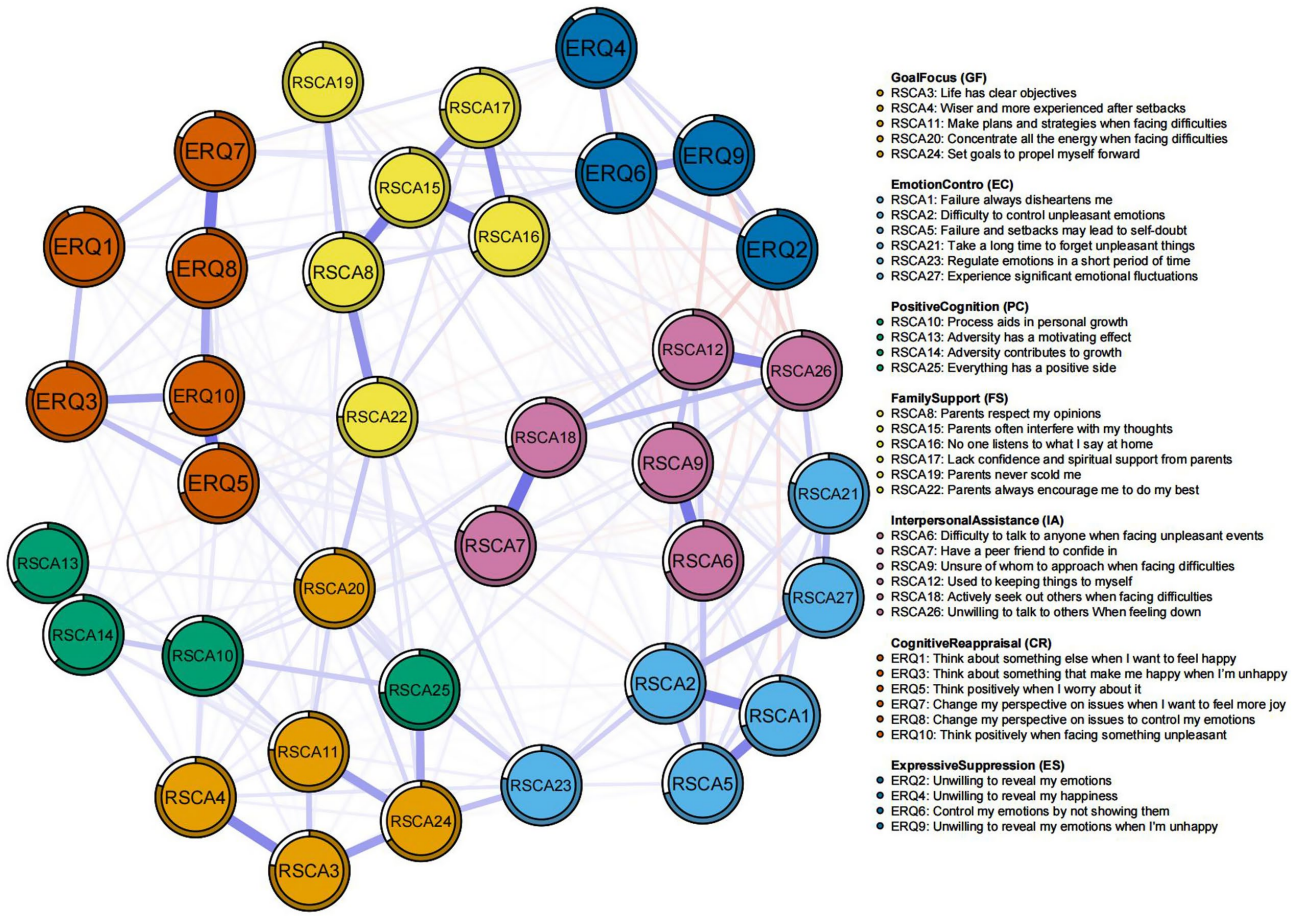
Network structure for the whole sample.

The network structure depicting the relationships between adolescents’ psychological resilience and emotion regulation, estimated using the EBICglasso model, is presented in Figure 1. Each node represents an individual questionnaire item. The network shows full interconnectivity with no isolated nodes.

#### 3.2.1 The characteristics of edges

The network theoretically could contain up to 666 possible edges, each representing a potential relationship between two distinct items. However, only 311 edges with weights ranging from −0.106 to 0.664 were retained as non-zero (Figure 1). Specifically, within the psychological resilience cluster, the strongest edge was PC_RSCA13 (“Adversity has a motivating effect”)-PC_RSCA14 (“Adversity contributes to growth”); within the emotion regulation cluster, it was CR_ERQ7 (“Change my perspective on issues when I want to feel more joy”)-CR_ERQ8 (“Change my perspective on issues to control my emotions”). Positive and negative associations between psychological resilience and emotion regulation were predominantly manifested through PC_RSCA25 (Everything has a positive side)-CR_ERQ5 (Think positively when I worry about it) and IA_RSCA26 (Unwilling to talk to others when feeling down)-ES_ERQ9 (Unwilling to reveal my emotions when I’m unhappy) (Supplementary Table S1).

#### 3.2.2 Node centrality indices

**Figure 2:**
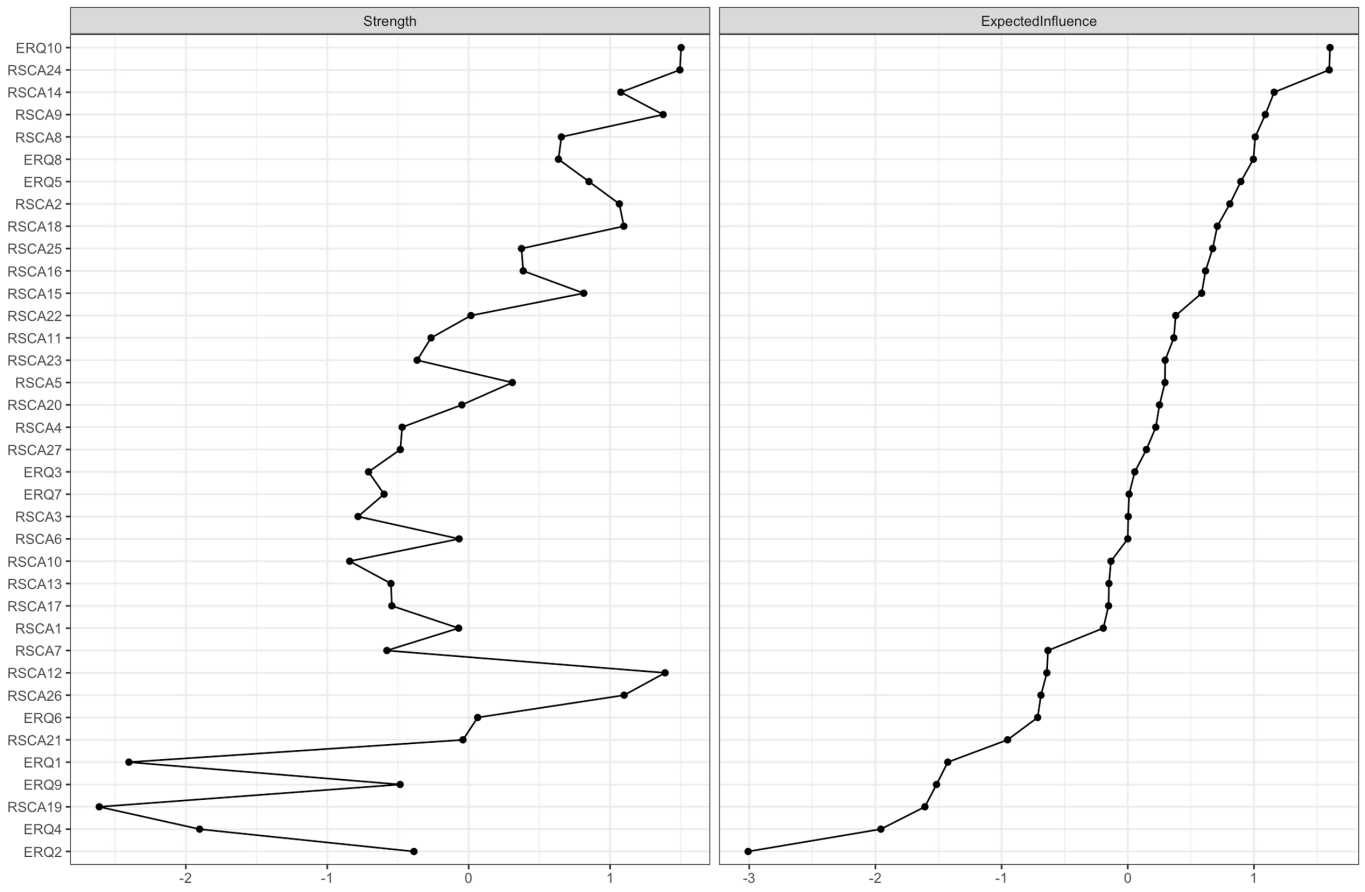
Centrality indices for the whole sample. The values plotted on the *x*-axis are standardized *z*-scores. Nodes are ordered by their EI.

The following results report the standardized *z*-scores of centrality indices, which are depicted in Figure 2. CR_ERQ10 showed the highest node strength (strength = 1.502), indicating the most direct connections to other nodes, followed by GF_RSCA24 (strength = 1.493) and IA_RSCA12 (strength = 1.389). Regarding EI, CR_ERQ10 also ranked highest (EI = 1.603), followed by GF_RSCA24 (EI = 1.597) and PC_RSCA14 (EI = 1.160). Both GF_RSCA24 (“Setting goals to propel myself forward”) and CR_ERQ10 (“Thinking positively when facing something unpleasant”) demonstrated notably high strength and EI values.

#### 3.2.3 Bridge centrality indices

**Figure 3:**
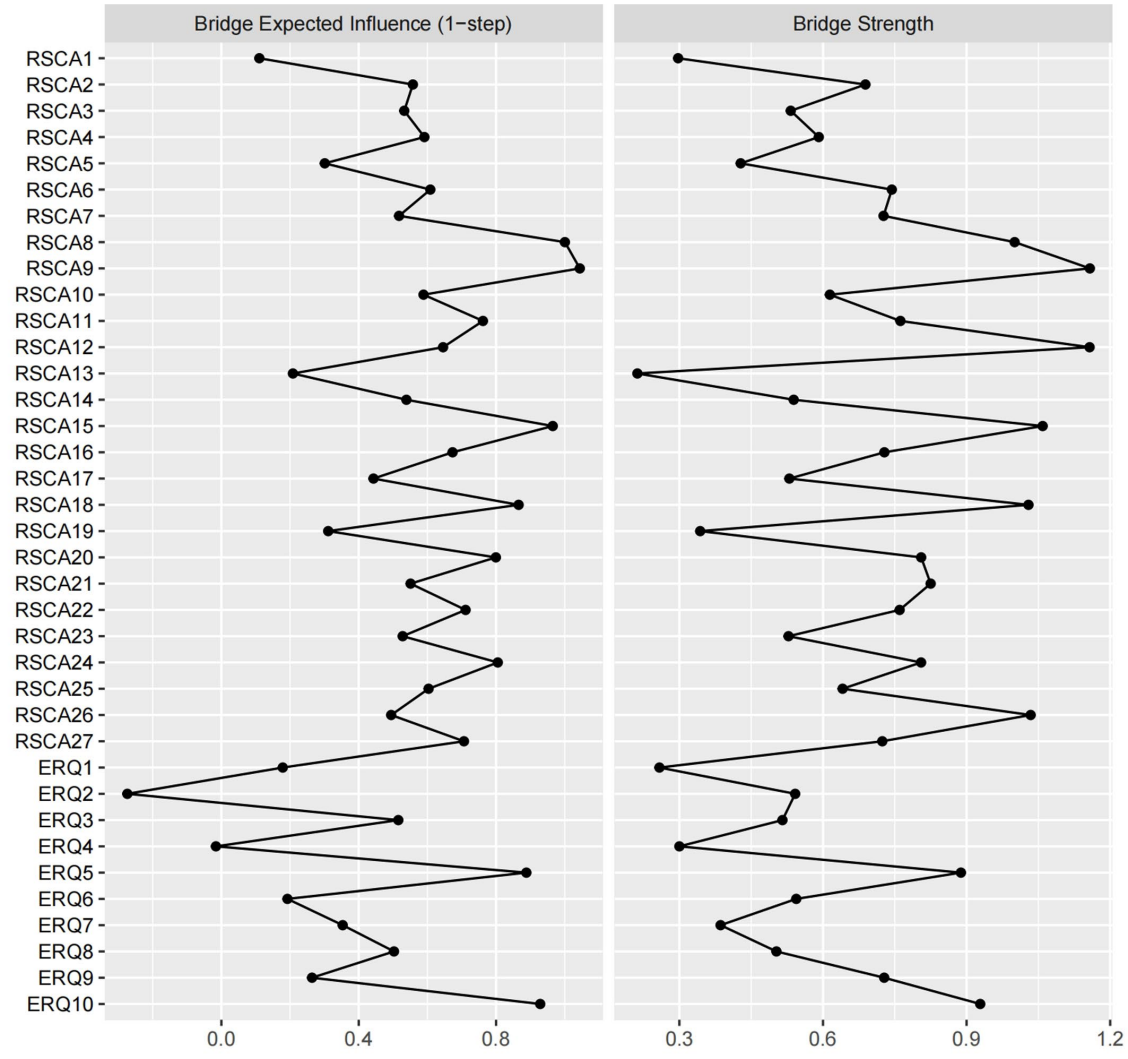
Bridge centrality indices for the whole sample. The values plotted on the *x*-axis are standardized *z*-scores.

The following results report the standardized *z*-scores of bridge centrality indices, which are depicted in Figure 3. Examination of the network structure (Figure 1) reveals that each of the seven clusters formed a stable and interconnected substructure. Based on the raw point estimates, nodes ranking in the top 20% for bridge expected influence (BEI) were: IA_RSCA9 (BEI = 1.044), FS_RSCA8 (BEI = 1.000), FS_RSCA15 (BEI = 0.965), CR_ERQ10 (BEI = 0.929), CR_ERQ5 (BEI = 0.888), IA_RSCA18 (BEI = 0.866), and GF_RSCA24 (BEI = 0.805). Similarly, nodes in the top 20% for raw bridge strength included: IA_RSCA9 (bridge strength = 1.158), IA_RSCA12 (bridge strength = 1.157), FS_RSCA15 (bridge strength = 1.059), IA_RSCA26 (bridge strength = 1.034), IA_RSCA18 (bridge strength = 1.029), FS_RSCA8 (bridge strength = 1.000), and CR_ERQ10 (bridge strength = 0.929).

Notably, within the psychological resilience cluster, IA_RSCA9 (“Unsure of whom to approach when facing difficulties”) exhibited the highest raw value for BEI and bridge strength. Within the emotion regulation cluster, CR_ERQ10 showed the highest raw value BEI and bridge strength. Together, these nodes represent the most critical bidirectional connectors between the two constructs.

### 3.3 Network stability

In the estimated network of adolescents’ psychological resilience and emotion regulation, edge weights demonstrated relatively narrow 95% confidence intervals, indicating acceptable estimation accuracy (Supplementary Figure S1). The stability of centrality indices was assessed using a case-dropping bootstrap approach. As subsample size decreased, the mean correlation of both strength and bridge strength indices between the original sample and subsamples diminished (Supplementary Figure S2). The correlation stability (CS) coefficients for strength and bridge strength were both 0.75, exceeding the recommended threshold of 0.50 and indicating robust stability.

### 3.4 Difference tests

Bootstrapped difference tests were conducted to compare edge weights and centrality indices. In the resulting difference plots, the matrix diagonal (comparing a node’s index with itself) represents theoretically zero differences. Although minor non-zero values may appear due to computational precision and bootstrap sampling variation, these diagonal elements are statistically uninformative and should be disregarded in interpretation. The off-diagonal black boxes represent statistically significant differences (*p* < 0.05) between two distinct edge weights or node centralities, while gray boxes represent non-significant differences (*p* > 0.05). The bootstrapped difference test identified the edge between PC_RSCA13 and PC_RSCA14 as the strongest, which was significantly stronger than all other edges (Supplementary Figure S3). Bootstrapped difference tests for node strength (Supplementary Figure S4) and expected influence (Supplementary Figure S5) consistently identified CR_ERQ10 and GF_RSCA24 as the nodes with the most statistically robust superiority, as their centrality indices were significantly higher than those of the vast majority of other nodes in the network. Furthermore, despite not having the highest raw value, PC_RSCA25 demonstrated significantly greater BEI than the majority of other nodes. This key result indicates that PC_RSCA25 is the most statistically robust and reliable connector between psychological resilience and emotion regulation (Supplementary Figures S6, S7).

### 3.5 Network comparison

**Figure 4:**
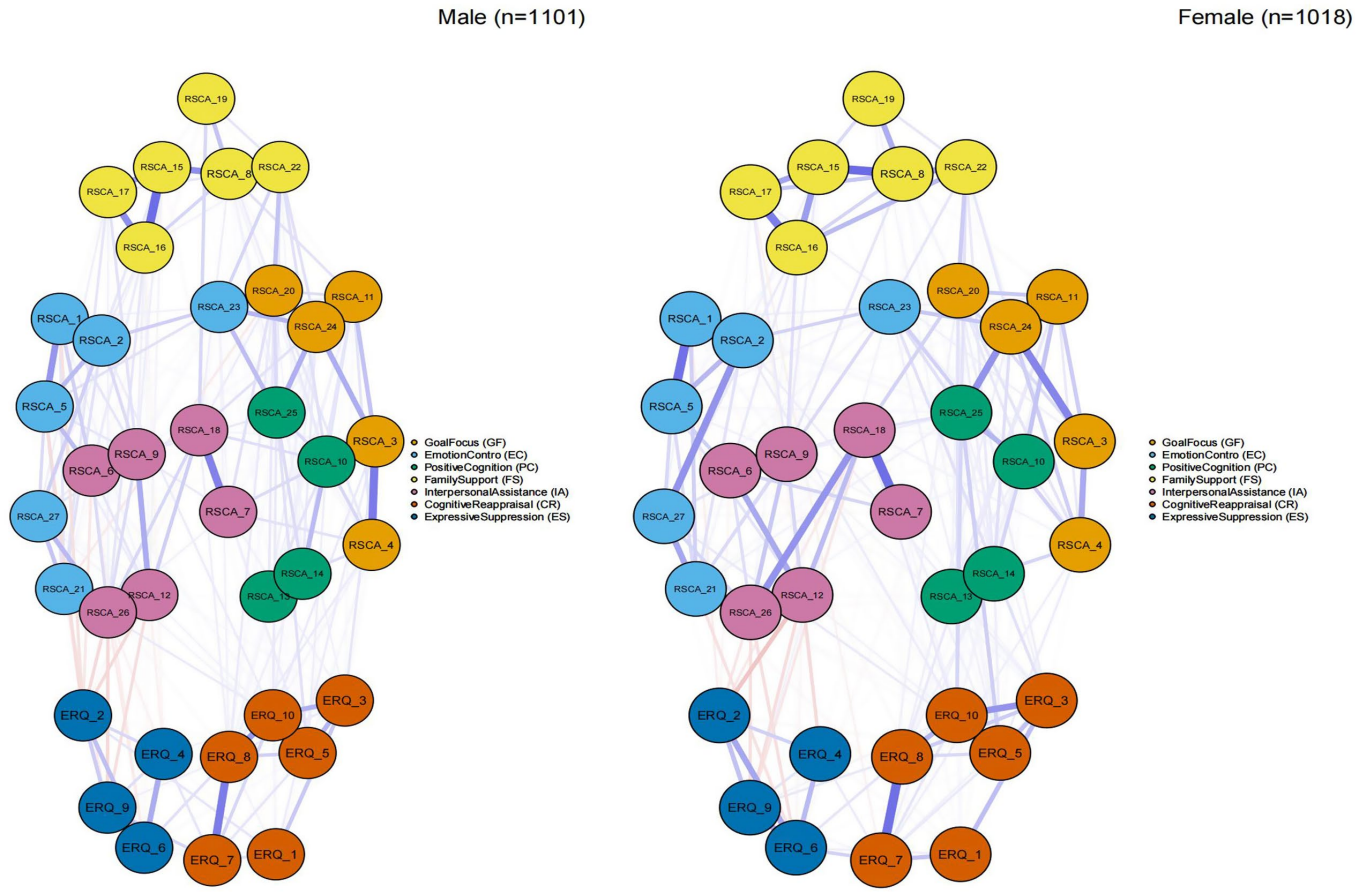
Network comparison between genders.

The network structures for male and female participants are depicted in Figure 4. The Network Comparison Test (NCT) revealed no significant difference in global strength between the female (strength = 17.073) and male (strength = 16.867) networks (*S* = 0.206, *p* = 0.682). However, a significant difference was found in the network invariance test (*M* = 0.151, *p* = 0.031), indicating a structural difference in how nodes are connected. Edge invariance tests showed that the majority of edges did not differ significantly between genders (*p* > 0.05), though three specific edges demonstrated significant differences (*p* < 0.05): PC_RSCA10-FS_RSCA17, IA_RSCA18- IA_RSCA26, EC_RSCA2-EC_RSCA27 (Supplementary Table S2). All centrality indices showed no significant differences between networks (all *p* > 0.05; Supplementary Table S3).

## 4 Discussion

This network analysis study yielded three principal findings regarding the relationship between psychological resilience and emotion regulation in Chinese early adolescents. First, the network structure was well-connected, with goal focus (e.g., “Setting goals to propel myself forward”) and cognitive reappraisal (e.g., “Thinking positively when facing something unpleasant”) identified as the most central nodes within their respective clusters. Second, bridge centrality analysis, refined by bootstrapped difference tests, identified the positive cognition item “Believing that everything has a positive side” (PC_RSCA25) as the most statistically robust bridge connecting the two constructs, highlighting positive cognitive appraisal as the paramount pathway of interconnection. Third, the network structure differed significantly by gender, with females exhibiting stronger connectivity, particularly within the psychological resilience cluster.

The results of the study on the boundary weights indicated that the most robust positive associations between psychological resilience and the emotion regulation network were identified between PC_RSCA25 and CR_ERQ5. This finding offers empirical support for the affect-regulation framework of psychological resilience (Troy and Mauss, 2011), which posits that the capacity to adaptively regulate emotions is a cornerstone of resilience. This implies that individuals with a positive cognitive disposition are more likely to employ cognitive reappraisal—an antecedent-focused strategy that reinterprets an emotional event’s meaning before the emotional response is fully generated (Gross, 1998). This early regulatory effort, as evidenced by neuroimaging studies, involves prefrontal cortical activation that dampens amygdala reactivity (Goldin et al., 2008), thereby effectively managing negative affect and preserving psychological resources. Consequently, this successful emotion regulation fosters positive adaptation, thereby enhancing psychological resilience. The most robust negative associations were identified between IA_RSCA26 and ES_ERQ9. This aligns with the theoretical understanding that social withdrawal and expressive suppression—a response-focused strategy that inhibits emotional expression after the emotion has arisen—can impede the social support-seeking that is crucial for resilience (Werner, 1993), while simultaneously increasing physiological arousal and cognitive load, as seen in heightened amygdala activity, thereby depleting resources and weakening psychological resilience.

Node centrality analysis identified “Setting goals to propel myself forward” (GF_RSCA24) and “Thinking positively when facing something unpleasant” (CR_ERQ10) as the most central nodes. This can be interpreted through the dual-process model of emotion regulation (Gyurak et al., 2011). Goal focus represents an explicit, top-down process for future-oriented planning, while cognitive reappraisal operates at the explicit-implicit interface. Their high centrality indicates they are key hubs coordinating regulatory efforts, whereby their activation likely propagates adaptive responses network-wide. This aligns with longitudinal findings on goal orientation (Werner and Smith, 1992) and Gross’s model emphasizing reappraisal’s role in well-being (Gross, 1998).

Bridge centrality analysis, refined by bootstrapped difference testing, demonstrated that positive cognition as the most robust and reliable bridge between the two constructs. An individual’s inherent belief that adversity can contain positive elements directly facilitates the employment of adaptive emotion regulation strategies, such as cognitive reappraisal, by providing the fundamental “raw material” for positive reinterpretation. This finding aligns with core resilience theories, which posit that the ability to find meaning and positive aspects in hardship is a hallmark of resilient functioning (Werner, 1993). Consequently, interventions aimed at enhancing psychological resilience by leveraging emotion regulation mechanisms should prioritize the cultivation of this positive cognitive style.

This study revealed nuanced gender differences in both scale scores and network architecture of psychological resilience and emotion regulation. Consistent with previous research across diverse populations (Lowe et al., 2021; Pan et al., 2024; Rasheed et al., 2022), male adolescents reported higher scores on most resilience dimensions. Furthermore, distinct patterns emerged in emotion regulation strategies: males reported greater use of cognitive reappraisal, while females scored higher on expressive suppression. This pattern contrasts with some Western findings but aligns with cultural models in East Asian contexts, where males may be socialized to use reappraisal for social harmony, and females may employ suppression as a form of relationship maintenance (Matsumoto et al., 2008). Crucially, network analysis provided a deeper structural explanation for these differences. The female network exhibited greater connectivity and stronger correlations, particularly within the psychological resilience cluster. Notably, this higher connectivity coexisted with females’ lower self-reported resilience scores—an apparent paradox that underscores qualitative differences in psychological architecture. This pattern suggests a more interdependent and integrated psychological architecture in females, where distress in one domain can propagate more readily through the dense network (Nolen-Hoeksema, 2012), a process facilitated by their relational self-construal (Cross and Madson, 1997) and intense focus on peer relationships (Rose and Rudolph, 2006). This readily transmission of distress potentially leads to lower global self-ratings of resilience. Conversely, the sparser connectivity in the male network might reflect a more compartmentalized structure, buffering the spread of distress and contributing to higher overall scale scores (Showers, 1992). This finding aligns with gender-role socialization theories, whereby females are often socialized to develop more interdependent coping systems, integrating social resources closely (Brody and Hall, 2008; Eagly and Wood, 2013), while males’ resilience networks may rely more on autonomous resources. Thus, interventions should be gender-specific: for females, leveraging interconnected social support; for males, enhancing individual regulatory skills while encouraging help-seeking.

Our findings significantly advance the affective-regulation theory of resilience by delineating its precise architecture at the item-level. We empirically identify positive cognition as the most robust bridge mechanism between resilience and emotion regulation—a specific pathway previously postulated but not quantitatively demonstrated. This network approach moves beyond broad dimension-level associations to reveal the exact components (PC_RSCA25, GF_RSCA24) and their dynamic interactions that operationalize resilience, thereby providing a mechanistic, culturally contextualized model for adolescent mental health in collectivistic societies.

Based on the network findings, a tiered intervention approach is recommended. Priority should be given to fortifying the most robust bridge, positive cognition (e.g., “believing everything has a positive side”), using CBT-based cognitive restructuring to foster finding value in adversity. Subsequently, SMART goal-setting exercises can strengthen the central node of goal focus. Modules targeting interpersonal assistance can be integrated secondarily. Implementing this prioritized sequence within school-based programs may most effectively enhance the resilience network by first securing its key connection to emotion regulation.

## 5 Limitation

There are some limitations in this study. Firstly, the limited sociodemographic information (e.g., socioeconomic status, family structure) constrains our ability to examine how these critical factors influence the network structure of psychological resilience and emotion regulation. Secondly, the cross-sectional design of this study precludes causal inferences about the dynamic interplay between the constructs. Thirdly, the reliance on self-report questionnaires may introduce subjectivity and recall bias. Finally, the sample, comprising early adolescents from one geographic region, may limit the generalizability of the findings to other age groups or cultural contexts.

## 6 Conclusion

In conclusion, this network analysis identified “Setting goals to propel myself forward” and “Thinking positively when facing something unpleasant” as central nodes, and “Believing everything has a positive side” as the key bridge between psychological resilience and emotion regulation. The female network showed stronger connectivity. Interventions should target these specific components—particularly fostering positive cognitive appraisal—to effectively enhance the adolescent mental health system.

## Data Availability

The raw data supporting the conclusions of this article will be made available by the authors, without undue reservation.
