# Supplementary figures and images for: The relationship between psychological resilience and emotion regulation in Chinese adolescents: a psychological network analysis
Source: Front Psychol. 2025 Nov 19;16:1552109. doi: 10.3389/fpsyg.2025.1552109 (PMC12672892; doi:10.3389/fpsyg.2025.1552109)

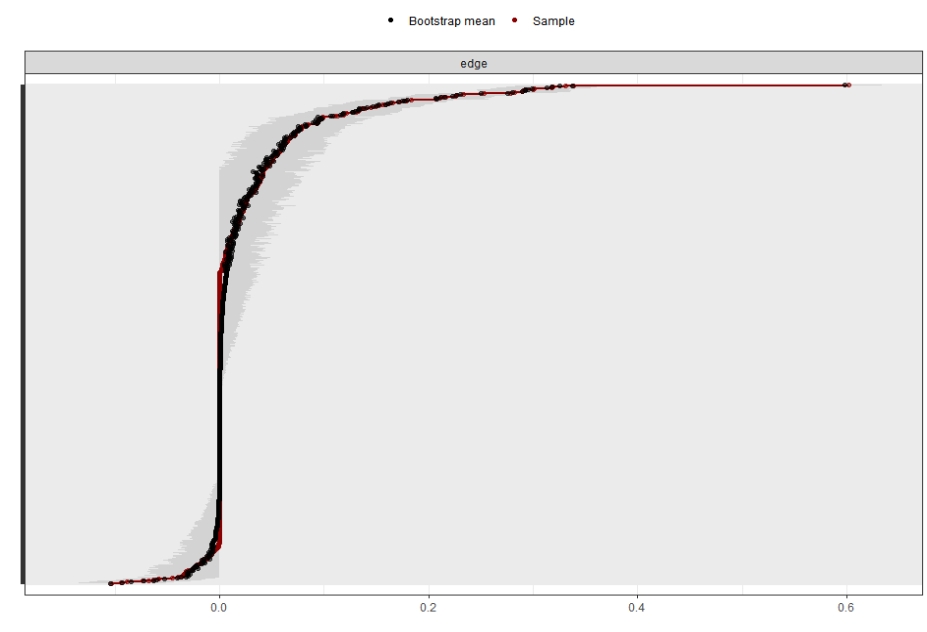

Supplement: Supplementary file 1 [file Image_1.jpeg]

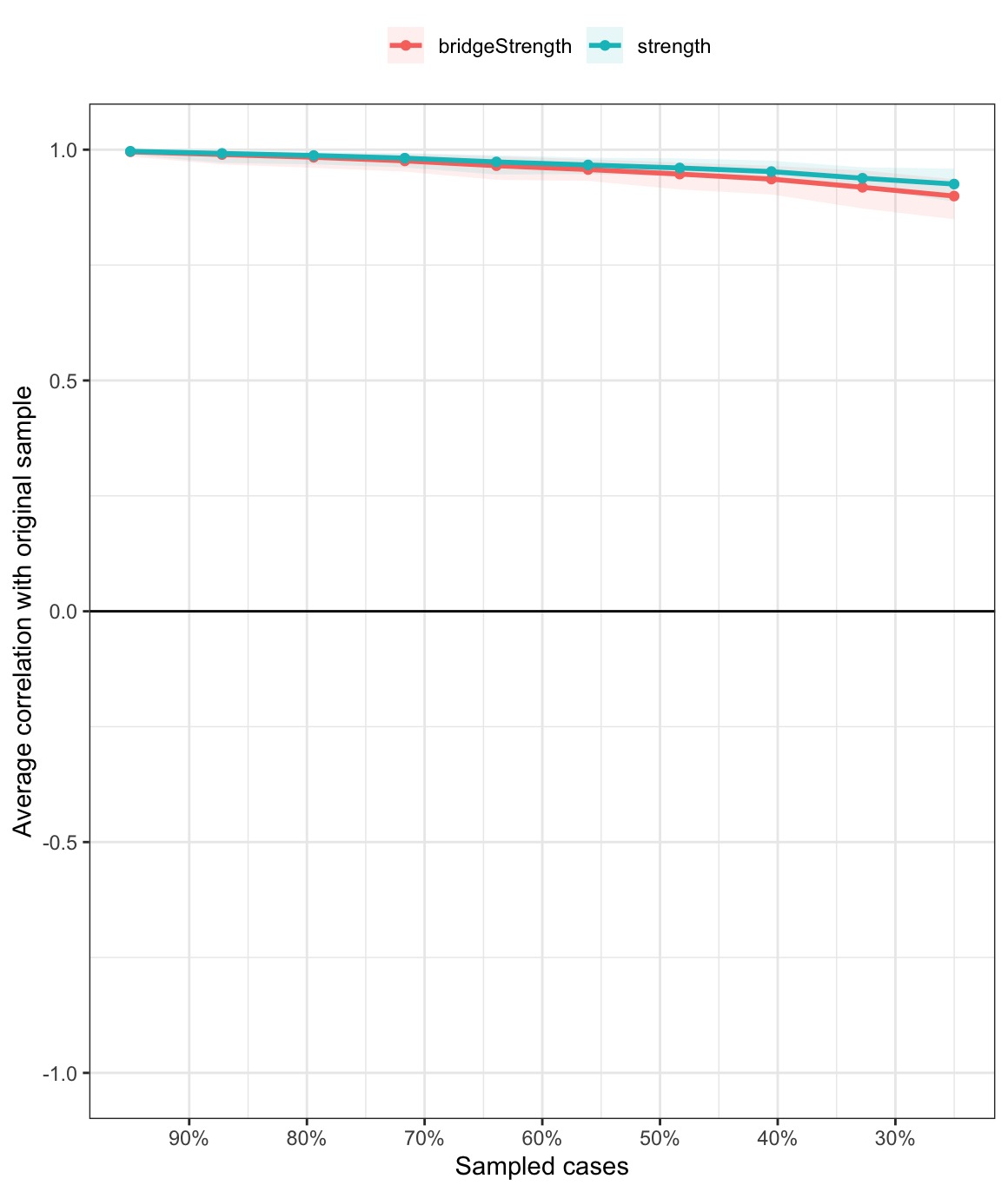

Supplement: Supplementary file 2 [file Image_2.jpeg]

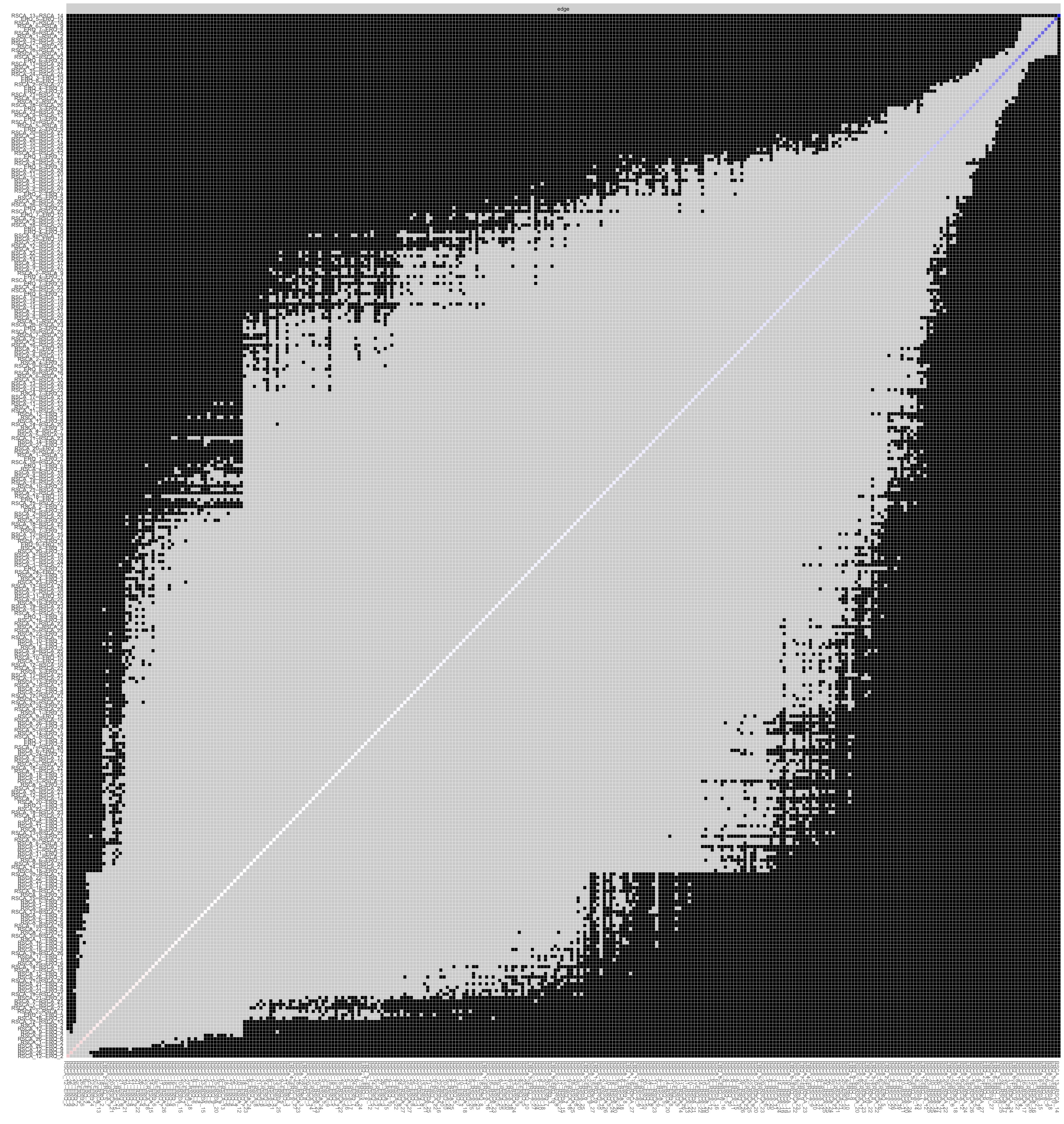

Supplement: Supplementary file 3 [file Image_3.jpeg]

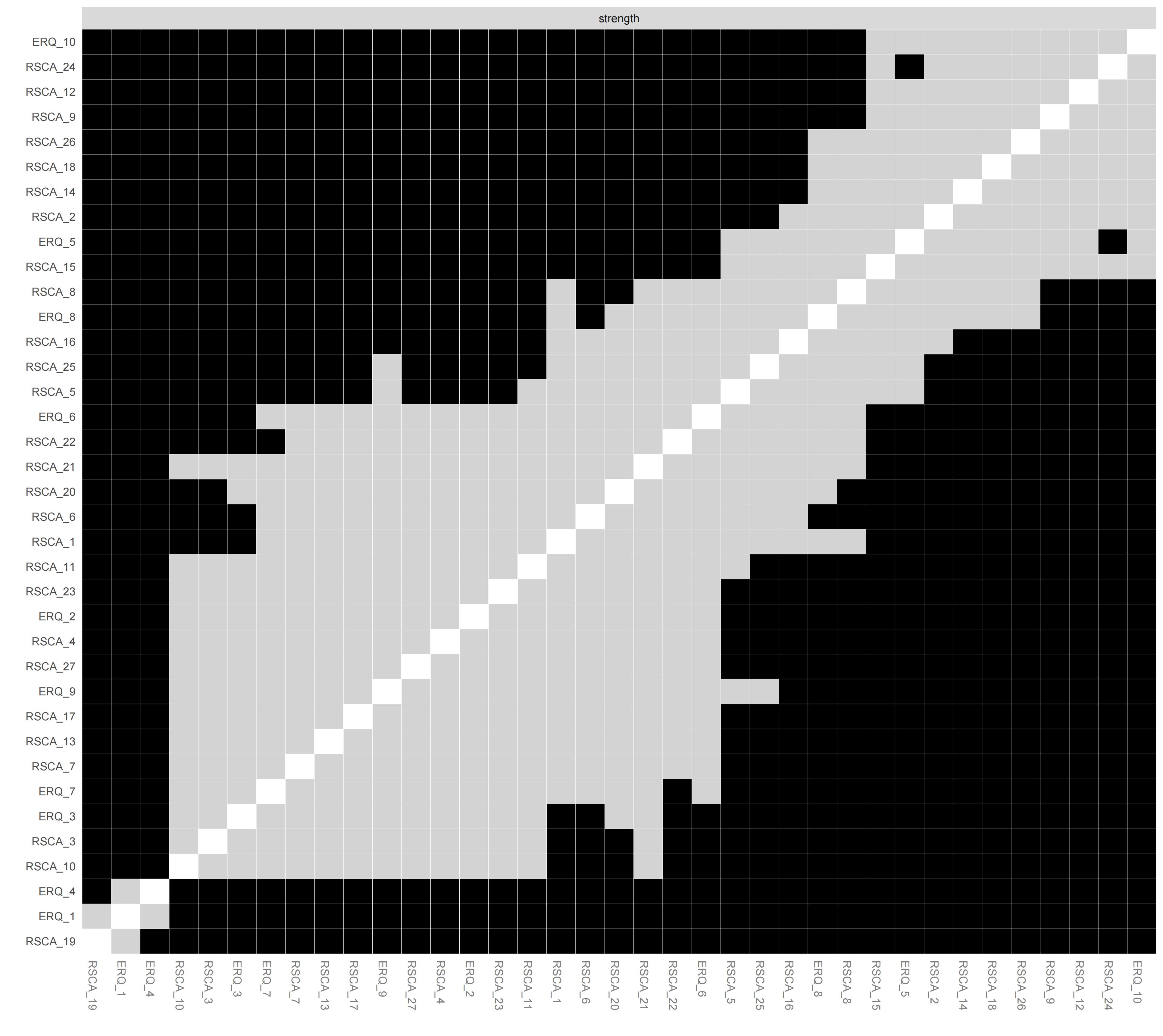

Supplement: Supplementary file 4 [file Image_4.jpeg]

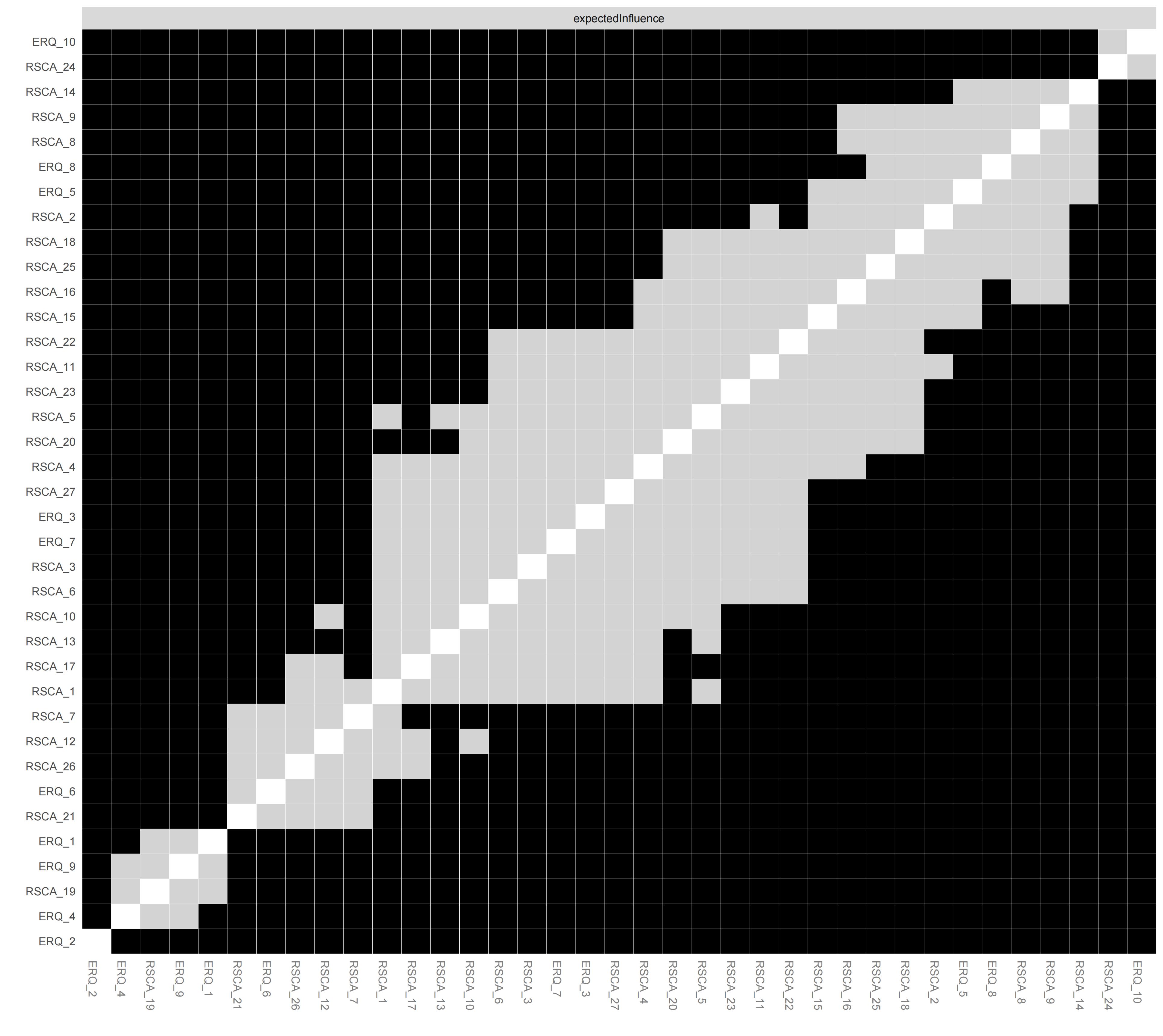

Supplement: Supplementary file 5 [file Image_5.jpeg]

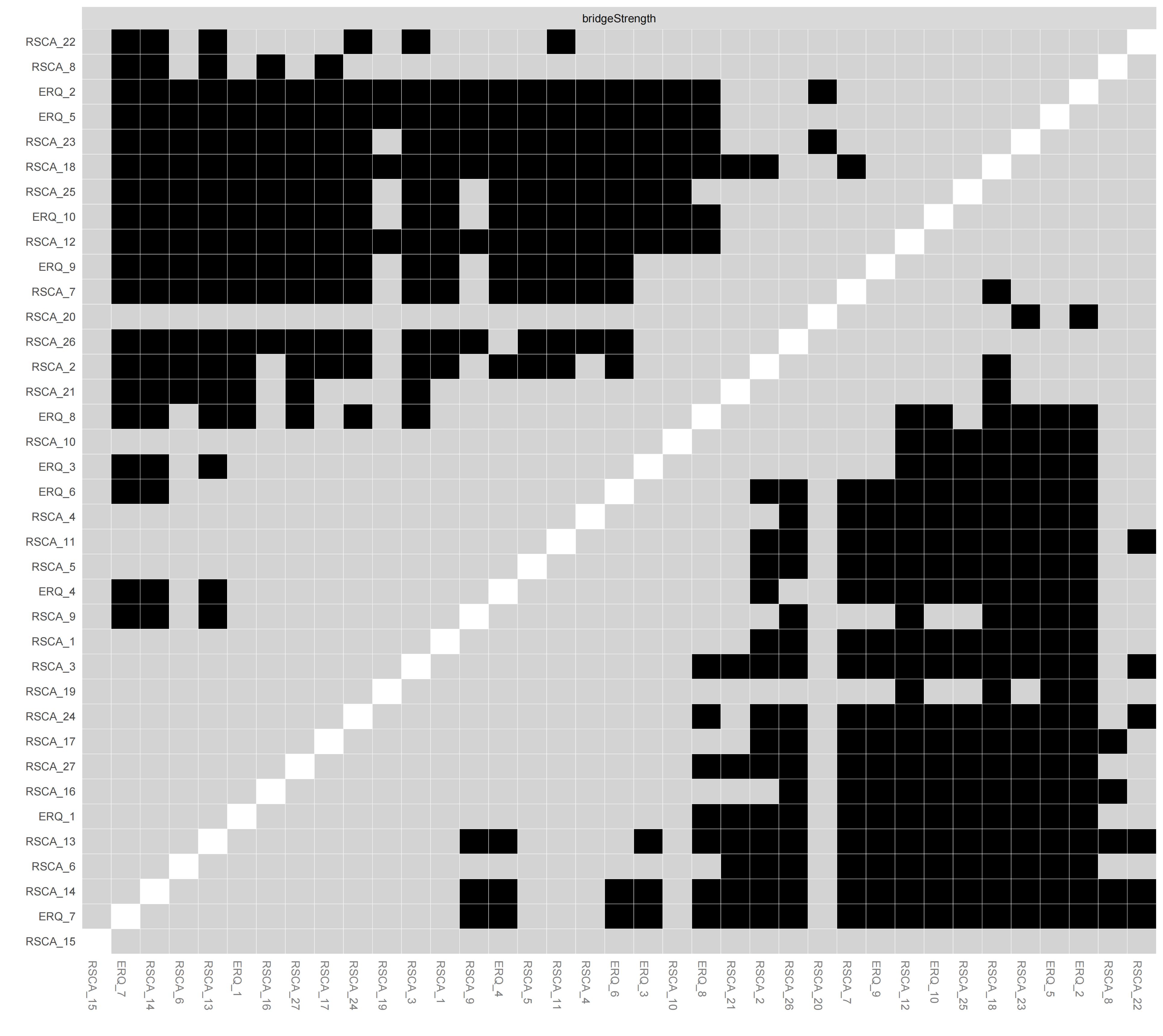

Supplement: Supplementary file 6 [file Image_6.jpeg]

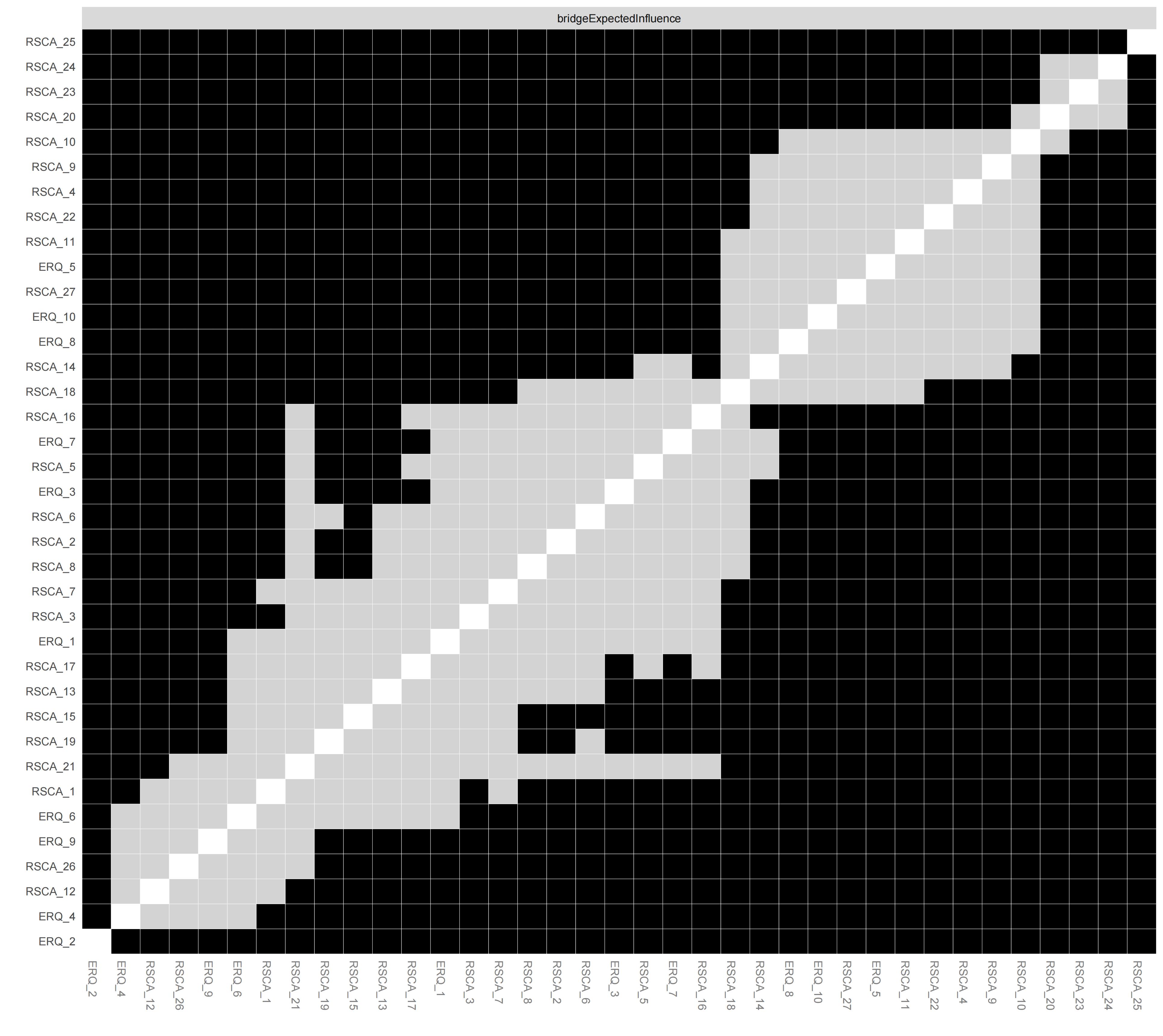

Supplement: Supplementary file 7 [file Image_7.jpeg]
