# Supplementary material for: The relationship between psychological resilience and emotion regulation in Chinese adolescents: a psychological network analysis
Source: Front Psychol. 2025 Nov 19;16:1552109. doi: 10.3389/fpsyg.2025.1552109 (PMC12672892; doi:10.3389/fpsyg.2025.1552109)
Supplement: Supplementary file 8 [file Table_1.docx]

**Table S1.** Edge weights for the whole network structure.

| Node1 | Node2 | weight |
| --- | --- | --- |
| RSCA_1 | RSCA_2 | 0.365145 |
| RSCA_1 | RSCA_5 | 0.323435222 |
| RSCA_1 | RSCA_6 | 0.053492462 |
| RSCA_1 | RSCA_7 | -0.012920827 |
| RSCA_1 | RSCA_9 | 0.037467187 |
| RSCA_1 | RSCA_10 | -0.012796278 |
| RSCA_1 | RSCA_11 | 0.014020932 |
| RSCA_1 | RSCA_12 | 0.009931339 |
| RSCA_1 | RSCA_18 | -0.033727036 |
| RSCA_1 | RSCA_19 | -0.004948549 |
| RSCA_1 | RSCA_21 | 0.088995449 |
| RSCA_1 | RSCA_22 | -0.026546332 |
| RSCA_1 | RSCA_23 | 0.015221825 |
| RSCA_1 | RSCA_24 | 0.036918513 |
| RSCA_1 | ERQ_1 | -0.003782053 |
| RSCA_1 | ERQ_2 | -0.075794678 |
| RSCA_1 | ERQ_5 | 0.015814916 |
| RSCA_1 | ERQ_6 | -0.003447932 |
| RSCA_2 | RSCA_5 | 0.159373185 |
| RSCA_2 | RSCA_6 | 0.003307512 |
| RSCA_2 | RSCA_7 | -0.051581306 |
| RSCA_2 | RSCA_9 | 0.072870729 |
| RSCA_2 | RSCA_11 | 0.021909833 |
| RSCA_2 | RSCA_15 | 0.042668949 |
| RSCA_2 | RSCA_16 | 0.016467663 |
| RSCA_2 | RSCA_18 | -0.038165247 |
| RSCA_2 | RSCA_21 | 0.07025322 |
| RSCA_2 | RSCA_22 | -0.013100159 |
| RSCA_2 | RSCA_23 | 0.119578356 |
| RSCA_2 | RSCA_25 | 0.029211777 |
| RSCA_2 | RSCA_27 | 0.209015132 |
| RSCA_2 | ERQ_1 | -0.024131143 |
| RSCA_2 | ERQ_2 | -0.004342124 |
| RSCA_2 | ERQ_3 | 0.005513985 |
| RSCA_2 | ERQ_6 | -0.007357377 |
| RSCA_2 | ERQ_9 | 0.062649744 |
| RSCA_2 | ERQ_10 | 0.06015182 |
| RSCA_3 | RSCA_4 | 0.334873289 |
| RSCA_3 | RSCA_7 | 0.013626905 |
| RSCA_3 | RSCA_9 | 0.003535478 |
| RSCA_3 | RSCA_11 | 0.129935351 |
| RSCA_3 | RSCA_18 | 0.014774272 |
| RSCA_3 | RSCA_20 | 0.01490433 |
| RSCA_3 | RSCA_24 | 0.235797055 |
| RSCA_3 | RSCA_27 | 0.01864862 |
| RSCA_3 | ERQ_1 | 0.023024469 |
| RSCA_3 | ERQ_3 | 0.042597428 |
| RSCA_3 | ERQ_10 | 0.011990521 |
| RSCA_4 | RSCA_7 | 0.046766551 |
| RSCA_4 | RSCA_8 | 0.020746566 |
| RSCA_4 | RSCA_10 | 0.072593225 |
| RSCA_4 | RSCA_11 | 0.05817064 |
| RSCA_4 | RSCA_14 | 0.098137179 |
| RSCA_4 | RSCA_20 | 0.054353366 |
| RSCA_4 | RSCA_21 | 0.000193433 |
| RSCA_4 | RSCA_22 | 0.006266956 |
| RSCA_4 | RSCA_23 | 0.060344997 |
| RSCA_4 | RSCA_24 | 0.010615591 |
| RSCA_4 | RSCA_27 | 0.003328539 |
| RSCA_4 | ERQ_3 | 0.025712654 |
| RSCA_4 | ERQ_5 | 0.046667194 |
| RSCA_4 | ERQ8 | 0.035074122 |
| RSCA_5 | RSCA_6 | 0.150662202 |
| RSCA_5 | RSCA_9 | 0.034462517 |
| RSCA_5 | RSCA_12 | 0.040590289 |
| RSCA_5 | RSCA_16 | 0.005528518 |
| RSCA_5 | RSCA_21 | 0.072932165 |
| RSCA_5 | RSCA_23 | 0.054152737 |
| RSCA_5 | RSCA_24 | 0.014287851 |
| RSCA_5 | RSCA_25 | 0.006613118 |
| RSCA_5 | RSCA_27 | 0.089055719 |
| RSCA_5 | ERQ_1 | -0.029649055 |
| RSCA_5 | ERQ_2 | -0.020944168 |
| RSCA_5 | ERQ_5 | 0.007079249 |
| RSCA_5 | ERQ_6 | -0.00610309 |
| RSCA_5 | ERQ_9 | -0.020420716 |
| RSCA_6 | RSCA_7 | 0.055458284 |
| RSCA_6 | RSCA_9 | 0.357940142 |
| RSCA_6 | RSCA_12 | 0.112509427 |
| RSCA_6 | RSCA_13 | -0.015767378 |
| RSCA_6 | RSCA_15 | 0.004162239 |
| RSCA_6 | RSCA_16 | 0.036237236 |
| RSCA_6 | RSCA_18 | 0.038939786 |
| RSCA_6 | RSCA_21 | 0.000270212 |
| RSCA_6 | RSCA_26 | 0.076489496 |
| RSCA_6 | ERQ_2 | -0.063611137 |
| RSCA_6 | ERQ_5 | 0.004017004 |
| RSCA_6 | ERQ_6 | -0.00591739 |
| RSCA_6 | ERQ_10 | 0.009780312 |
| RSCA_7 | RSCA_8 | 0.019584361 |
| RSCA_7 | RSCA_9 | 0.084072312 |
| RSCA_7 | RSCA_10 | 0.071409171 |
| RSCA_7 | RSCA_11 | 0.007104359 |
| RSCA_7 | RSCA_15 | -0.056375567 |
| RSCA_7 | RSCA_18 | 0.370133559 |
| RSCA_7 | RSCA_20 | 0.004356703 |
| RSCA_7 | RSCA_21 | -0.057885811 |
| RSCA_7 | RSCA_24 | 0.005322392 |
| RSCA_7 | RSCA_26 | 0.037591923 |
| RSCA_7 | RSCA_27 | -0.011009532 |
| RSCA_7 | ERQ_1 | 0.029156556 |
| RSCA_7 | ERQ_3 | 0.056706454 |
| RSCA_7 | ERQ_4 | -0.008259069 |
| RSCA_7 | ERQ_5 | 0.03764956 |
| RSCA_7 | ERQ_7 | 0.004081708 |
| RSCA_8 | RSCA_10 | 0.048412193 |
| RSCA_8 | RSCA_11 | 0.053915251 |
| RSCA_8 | RSCA_14 | 0.002610841 |
| RSCA_8 | RSCA_15 | 0.324800152 |
| RSCA_8 | RSCA_16 | 0.084263202 |
| RSCA_8 | RSCA_17 | 0.069428185 |
| RSCA_8 | RSCA_18 | 0.004918024 |
| RSCA_8 | RSCA_19 | 0.173220025 |
| RSCA_8 | RSCA_22 | 0.278838825 |
| RSCA_8 | RSCA_25 | 0.005880043 |
| RSCA_8 | ERQ_3 | 0.028861488 |
| RSCA_8 | ERQ_5 | 0.012764609 |
| RSCA_9 | RSCA_12 | 0.159843473 |
| RSCA_9 | RSCA_15 | 0.052411522 |
| RSCA_9 | RSCA_16 | 0.051590555 |
| RSCA_9 | RSCA_17 | 0.070805286 |
| RSCA_9 | RSCA_18 | 0.015545485 |
| RSCA_9 | RSCA_21 | 0.039785456 |
| RSCA_9 | RSCA_22 | 0.017841742 |
| RSCA_9 | RSCA_25 | 0.053794679 |
| RSCA_9 | RSCA_26 | 0.081787113 |
| RSCA_9 | RSCA_27 | 0.058360686 |
| RSCA_9 | ERQ_4 | -0.048734703 |
| RSCA_9 | ERQ_6 | -0.004582392 |
| RSCA_9 | ERQ_9 | -0.005694668 |
| RSCA_9 | ERQ_10 | 0.011870535 |
| RSCA_10 | RSCA_11 | 0.106077308 |
| RSCA_10 | RSCA_12 | -0.040427982 |
| RSCA_10 | RSCA_13 | 0.057754634 |
| RSCA_10 | RSCA_14 | 0.130541738 |
| RSCA_10 | RSCA_15 | -0.038991939 |
| RSCA_10 | RSCA_18 | 0.067510501 |
| RSCA_10 | RSCA_20 | 0.056976561 |
| RSCA_10 | RSCA_22 | 0.037662221 |
| RSCA_10 | RSCA_25 | 0.124506327 |
| RSCA_10 | RSCA_27 | 0.008893816 |
| RSCA_10 | ERQ_1 | 0.007363071 |
| RSCA_10 | ERQ_2 | 0.003955892 |
| RSCA_10 | ERQ_5 | 0.02813383 |
| RSCA_10 | ERQ_10 | 0.017054389 |
| RSCA_11 | RSCA_13 | 0.038771944 |
| RSCA_11 | RSCA_14 | 0.039443902 |
| RSCA_11 | RSCA_18 | 0.010276859 |
| RSCA_11 | RSCA_20 | 0.104914702 |
| RSCA_11 | RSCA_21 | 0.003640677 |
| RSCA_11 | RSCA_23 | 0.025295972 |
| RSCA_11 | RSCA_24 | 0.245005248 |
| RSCA_11 | RSCA_25 | 0.013461519 |
| RSCA_11 | ERQ_1 | 0.005331309 |
| RSCA_11 | ERQ_4 | 0.011938321 |
| RSCA_11 | ERQ8 | 0.04162314 |
| RSCA_11 | ERQ_10 | 0.014001873 |
| RSCA_12 | RSCA_13 | -0.042638526 |
| RSCA_12 | RSCA_15 | 0.028328204 |
| RSCA_12 | RSCA_16 | 0.021234276 |
| RSCA_12 | RSCA_18 | 0.163797025 |
| RSCA_12 | RSCA_19 | -0.002563529 |
| RSCA_12 | RSCA_21 | 0.083021243 |
| RSCA_12 | RSCA_26 | 0.309174906 |
| RSCA_12 | ERQ_2 | -0.104101624 |
| RSCA_12 | ERQ_4 | -0.042255144 |
| RSCA_12 | ERQ_5 | 0.052965557 |
| RSCA_12 | ERQ_6 | -0.018428434 |
| RSCA_12 | ERQ_9 | -0.098629745 |
| RSCA_12 | ERQ_10 | 0.002487007 |
| RSCA_13 | RSCA_14 | 0.664314757 |
| RSCA_13 | RSCA_16 | -0.000378886 |
| RSCA_13 | RSCA_17 | -0.01563405 |
| RSCA_13 | RSCA_20 | 0.051252667 |
| RSCA_13 | RSCA_24 | 0.016242547 |
| RSCA_13 | RSCA_26 | -0.003453262 |
| RSCA_13 | ERQ_2 | -0.005538241 |
| RSCA_13 | ERQ_7 | 0.012760096 |
| RSCA_13 | ERQ8 | 0.010649001 |
| RSCA_14 | RSCA_15 | -0.025900745 |
| RSCA_14 | RSCA_20 | 0.036395356 |
| RSCA_14 | RSCA_21 | 0.00015164 |
| RSCA_14 | RSCA_22 | 0.047121654 |
| RSCA_14 | RSCA_24 | 0.06089857 |
| RSCA_14 | RSCA_25 | 0.066155782 |
| RSCA_14 | ERQ_2 | -0.00282302 |
| RSCA_14 | ERQ_4 | -0.008942747 |
| RSCA_14 | ERQ_5 | 0.007445204 |
| RSCA_14 | ERQ8 | 0.034587467 |
| RSCA_15 | RSCA_16 | 0.342414019 |
| RSCA_15 | RSCA_17 | 0.220694484 |
| RSCA_15 | RSCA_19 | 0.076078379 |
| RSCA_15 | RSCA_20 | -0.030426142 |
| RSCA_15 | RSCA_21 | 0.005996649 |
| RSCA_15 | RSCA_23 | -0.0128826 |
| RSCA_15 | RSCA_27 | 0.022295077 |
| RSCA_16 | RSCA_17 | 0.291080279 |
| RSCA_16 | RSCA_21 | 0.036975438 |
| RSCA_16 | RSCA_22 | 0.08277307 |
| RSCA_16 | RSCA_26 | 0.049289477 |
| RSCA_16 | RSCA_27 | 0.030397168 |
| RSCA_16 | ERQ_4 | -0.023932674 |
| RSCA_16 | ERQ_6 | -0.01259674 |
| RSCA_17 | RSCA_18 | -0.018221225 |
| RSCA_17 | RSCA_21 | 0.061290121 |
| RSCA_17 | RSCA_22 | 0.0888324 |
| RSCA_17 | RSCA_25 | -0.002031867 |
| RSCA_17 | RSCA_27 | 0.036480404 |
| RSCA_17 | ERQ_4 | -0.013952175 |
| RSCA_17 | ERQ_7 | -0.026646083 |
| RSCA_18 | RSCA_19 | 0.084361574 |
| RSCA_18 | RSCA_20 | 0.099580749 |
| RSCA_18 | RSCA_22 | 0.017812777 |
| RSCA_18 | RSCA_23 | 0.009619904 |
| RSCA_18 | RSCA_24 | 0.038624846 |
| RSCA_18 | RSCA_25 | 0.035028272 |
| RSCA_18 | RSCA_26 | 0.183131797 |
| RSCA_18 | ERQ_1 | 0.007685921 |
| RSCA_18 | ERQ_2 | -0.031787012 |
| RSCA_18 | ERQ_4 | -0.001714808 |
| RSCA_18 | ERQ_6 | -0.003579391 |
| RSCA_18 | ERQ8 | 0.024434389 |
| RSCA_18 | ERQ_9 | -0.004415562 |
| RSCA_18 | ERQ_10 | 0.019728795 |
| RSCA_19 | RSCA_20 | 0.040070932 |
| RSCA_19 | RSCA_21 | -0.047798694 |
| RSCA_19 | RSCA_22 | 0.094997896 |
| RSCA_19 | RSCA_23 | 0.020684619 |
| RSCA_19 | RSCA_26 | -0.048687535 |
| RSCA_19 | ERQ_5 | 0.01788533 |
| RSCA_20 | RSCA_21 | -0.056998991 |
| RSCA_20 | RSCA_22 | 0.152583064 |
| RSCA_20 | RSCA_23 | 0.081208261 |
| RSCA_20 | RSCA_24 | 0.091945435 |
| RSCA_20 | RSCA_25 | 0.063101988 |
| RSCA_20 | RSCA_26 | -0.010480475 |
| RSCA_20 | ERQ_1 | 0.001231245 |
| RSCA_20 | ERQ_7 | 0.020371559 |
| RSCA_20 | ERQ8 | 0.02158295 |
| RSCA_20 | ERQ_10 | 0.045893859 |
| RSCA_21 | RSCA_22 | -0.061887681 |
| RSCA_21 | RSCA_23 | 0.088645952 |
| RSCA_21 | RSCA_26 | 0.033834379 |
| RSCA_21 | RSCA_27 | 0.185764733 |
| RSCA_21 | ERQ_2 | -0.017582114 |
| RSCA_21 | ERQ_5 | 0.001318423 |
| RSCA_21 | ERQ_6 | -0.024786732 |
| RSCA_21 | ERQ_9 | -0.037324084 |
| RSCA_21 | ERQ_10 | 0.061348814 |
| RSCA_22 | RSCA_23 | 0.102354069 |
| RSCA_22 | RSCA_24 | 0.069734786 |
| RSCA_22 | RSCA_25 | 0.059009423 |
| RSCA_22 | ERQ_3 | 0.000941192 |
| RSCA_22 | ERQ_4 | -0.0046329 |
| RSCA_23 | RSCA_24 | 0.155512764 |
| RSCA_23 | RSCA_25 | 0.12798308 |
| RSCA_23 | ERQ_3 | 0.017122352 |
| RSCA_23 | ERQ_5 | 0.016232861 |
| RSCA_23 | ERQ8 | 0.02319476 |
| RSCA_23 | ERQ_9 | 0.041883331 |
| RSCA_24 | RSCA_25 | 0.246542737 |
| RSCA_24 | ERQ_7 | 0.005513531 |
| RSCA_24 | ERQ8 | 0.013705438 |
| RSCA_24 | ERQ_10 | 0.010691594 |
| RSCA_25 | ERQ_4 | -0.003617321 |
| RSCA_25 | ERQ_5 | 0.084289613 |
| RSCA_25 | ERQ_6 | -0.037996957 |
| RSCA_25 | ERQ8 | 0.013807537 |
| RSCA_25 | ERQ_10 | 0.077132869 |
| RSCA_26 | RSCA_27 | 0.167758342 |
| RSCA_26 | ERQ_2 | -0.073418865 |
| RSCA_26 | ERQ_3 | 0.011492568 |
| RSCA_26 | ERQ_4 | -0.010443944 |
| RSCA_26 | ERQ_6 | -0.070329988 |
| RSCA_26 | ERQ_9 | -0.106173812 |
| RSCA_27 | ERQ_1 | -0.004154433 |
| RSCA_27 | ERQ_3 | 0.00677438 |
| RSCA_27 | ERQ_5 | 0.031721361 |
| RSCA_27 | ERQ8 | 0.016395146 |
| ERQ_1 | ERQ_2 | 0.05220988 |
| ERQ_1 | ERQ_3 | 0.170431398 |
| ERQ_1 | ERQ_4 | 0.020509358 |
| ERQ_1 | ERQ_6 | 0.032888739 |
| ERQ_1 | ERQ_7 | 0.128006897 |
| ERQ_1 | ERQ_10 | 0.039785424 |
| ERQ_2 | ERQ_4 | 0.100793917 |
| ERQ_2 | ERQ_5 | -0.047658363 |
| ERQ_2 | ERQ_6 | 0.187693458 |
| ERQ_2 | ERQ_9 | 0.151036902 |
| ERQ_3 | ERQ_4 | 0.023916755 |
| ERQ_3 | ERQ_5 | 0.169857843 |
| ERQ_3 | ERQ_7 | 0.013586765 |
| ERQ_3 | ERQ8 | 0.076119666 |
| ERQ_3 | ERQ_10 | 0.21987651 |
| ERQ_4 | ERQ_5 | 0.015897937 |
| ERQ_4 | ERQ_6 | 0.189969476 |
| ERQ_4 | ERQ_7 | 0.078144046 |
| ERQ_4 | ERQ_9 | 0.083133348 |
| ERQ_5 | ERQ_6 | 0.049639066 |
| ERQ_5 | ERQ_7 | 0.068887173 |
| ERQ_5 | ERQ8 | 0.08997435 |
| ERQ_5 | ERQ_10 | 0.361050628 |
| ERQ_6 | ERQ_7 | 0.061479358 |
| ERQ_6 | ERQ8 | 0.091676607 |
| ERQ_6 | ERQ_9 | 0.245834266 |
| ERQ_7 | ERQ8 | 0.362319071 |
| ERQ_7 | ERQ_9 | 0.069109052 |
| ERQ_7 | ERQ_10 | 0.064686573 |
| ERQ8 | ERQ_9 | 0.050019297 |
| ERQ8 | ERQ_10 | 0.224718598 |
| ERQ_9 | ERQ_10 | 0.042612082 |
