# Supplementary material for: The relationship between psychological resilience and emotion regulation in Chinese adolescents: a psychological network analysis
Source: Front Psychol. 2025 Nov 19;16:1552109. doi: 10.3389/fpsyg.2025.1552109 (PMC12672892; doi:10.3389/fpsyg.2025.1552109)
Supplement: Supplementary file 9 [file Table_2.docx]

**Table S2.** The validity results of the Edge Invariance Test.

| Item | Item | p-value | Absolute difference |
| --- | --- | --- | --- |
| PC-RSCA_10 | FS-RSCA_17 | 0.000 | 0.007 |
| IA-RSCA_18 | IA-RSCA_26 | 0.000 | 0.151 |
| EC-RSCA_2 | EC-RSCA_27 | 0.000 | 0.136 |
