# Supplementary material for: The relationship between psychological resilience and emotion regulation in Chinese adolescents: a psychological network analysis
Source: Front Psychol. 2025 Nov 19;16:1552109. doi: 10.3389/fpsyg.2025.1552109 (PMC12672892; doi:10.3389/fpsyg.2025.1552109)
Supplement: Supplementary file 10 [file Table_3.docx]

**Table S3.** The validity results of Centrality Invariance Test.

| Node | Strength | Expected  Influence |
| --- | --- | --- |
| RSCA_1 | 0.956 | 0.956 |
| RSCA_2 | 0.956 | 0.956 |
| RSCA_3 | 0.932 | 0.932 |
| RSCA_4 | 0.956 | 0.956 |
| RSCA_5 | 1.000 | 0.897 |
| RSCA_6 | 0.956 | 0.932 |
| RSCA_7 | 0.932 | 0.932 |
| RSCA_8 | 0.932 | 0.932 |
| RSCA_9 | 0.956 | 0.956 |
| RSCA_10 | 0.932 | 0.932 |
| RSCA_11 | 0.932 | 0.932 |
| RSCA_12 | 0.897 | 0.932 |
| RSCA_13 | 0.956 | 0.956 |
| RSCA_14 | 0.956 | 0.956 |
| RSCA_15 | 0.932 | 0.956 |
| RSCA_16 | 0.956 | 0.956 |
| RSCA_17 | 0.897 | 0.956 |
| RSCA_18 | 0.914 | 0.956 |
| RSCA_19 | 0.966 | 0.932 |
| RSCA_20 | 0.932 | 0.956 |
| RSCA_21 | 0.956 | 0.932 |
| RSCA_22 | 0.932 | 0.956 |
| RSCA_23 | 0.956 | 0.956 |
| RSCA_24 | 0.956 | 0.956 |
| RSCA_25 | 0.888 | 0.932 |
| RSCA_26 | 0.956 | 0.932 |
| RSCA_27 | 0.897 | 0.932 |
| ERQ_1 | 0.956 | 0.956 |
| ERQ_2 | 0.932 | 0.914 |
| ERQ_3 | 0.897 | 0.897 |
| ERQ_4 | 1.000 | 0.909 |
| ERQ_5 | 0.956 | 0.956 |
| ERQ_6 | 0.932 | 0.956 |
| ERQ_7 | 0.897 | 0.897 |
| ERQ_8 | 0.897 | 0.897 |
| ERQ_9 | 0.932 | 0.932 |
| ERQ_10 | 0.956 | 0.956 |
